# Correction for retest effects across repeated measures of cognitive functioning: a longitudinal cohort study of postoperative delirium

**DOI:** 10.1186/s12874-018-0530-x

**Published:** 2018-07-03

**Authors:** Annie M. Racine, Yun Gou, Tamara G. Fong, Edward R. Marcantonio, Eva M. Schmitt, Thomas G. Travison, Sharon K. Inouye, Richard N. Jones

**Affiliations:** 1 Aging Brain Center, Institute for Aging Research, Hebrew SeniorLife, 1200 Centre St, Boston, MA 02131 USA; 2 Harvard Medical School, Boston, MA USA; 3 Athinoula A. Martinos Center for Biomedical Imaging, Massachusetts General Hospital, Boston, MA USA; 4 Department of Neurology, Beth Israel Deaconess Medical Center, Boston, MA USA; 5 Department of Medicine, Beth Israel Deaconess Medical Center, Boston, MA USA; 6 Department of Psychiatry and Human Behavior and Neurology, Brown University Warren Alpert Medical School, Providence, RI USA

**Keywords:** Retest, Practice, Surgery, Delirium, Cognitive decline, Repeated measures, Post-operative

## Abstract

**Background:**

Few studies have compared methods to correct for retest effects or practice effects in settings where an acute event could influence test performance, such as major surgery. Our goal in this study was to evaluate the use of different methods to correct for the effects of practice or retest on repeated test administration in the context of an observational study of older adults undergoing elective surgery.

**Methods:**

In a cohort of older surgical patients (*N* = 560) and a non-surgical comparison group (*N* = 118), we compared changes on repeated cognitive testing using a summary measure of general cognitive performance (GCP) between patients who developed post-operative delirium and those who did not. Surgical patients were evaluated pre-operatively and at 1, 2, 6, 12, and 18 months following surgery. Inferences from linear mixed effects models using four approaches were compared: 1) no retest correction, 2) mean-difference correction, 3) predicted-difference correction, and 4) model-based correction.

**Results:**

Using Approaches 1 or 4, which use uncorrected data, both surgical groups appeared to improve or remain stable after surgery. In contrast, Approaches 2 and 3, which dissociate retest and surgery effects by using retest-adjusted GCP scores, revealed an acute decline in performance in both surgical groups followed by a recovery to baseline. Relative differences between delirium groups were generally consistent across all approaches: the delirium group showed greater short- and longer-term decline compared to the group without delirium, although differences were attenuated after 2 months. Standard errors and model fit were also highly consistent across approaches.

**Conclusion:**

All four approaches would lead to nearly identical inferences regarding relative mean differences between groups experiencing a key post-operative outcome (delirium) but produced qualitatively different impressions of absolute performance differences following surgery. Each of the four retest correction approaches analyzed in this study has strengths and weakness that should be evaluated in the context of future studies. Retest correction is critical for interpretation of absolute cognitive performance measured over time and, consequently, for advancing our understanding of the effects of exposures such as surgery, hospitalization, acute illness, and delirium.

**Electronic supplementary material:**

The online version of this article (10.1186/s12874-018-0530-x) contains supplementary material, which is available to authorized users.

## Background

Studies of within-individual change in cognitive performance are critical to advancing our understanding of the impact of aging and disease on cognitive functioning. However, repeated test administration can result in observed gains in performance that may be due to familiarity with test content or context rather than true differences in underlying cognitive ability. These spurious gains, referred to as practice or retest effects, may be due to familiarity with the testing situation, reduced anxiety, or changes in the environment [1]. Retest effects are observed across a variety of cognitive domains [2, 3] and can last for several years [1]. The magnitude of decline attributable to aging or age-related disorders may be underestimated in longitudinal studies if practice or retest effects are not considered, and may even erroneously suggest longitudinal gains in performance [1, 4, 5].

Although multiple methods have been proposed to correct for retest effects in longitudinal studies, there is no clear consensus on the best approach [6]. Moreover, the ability to measure or adjust for practice or retest effects is further complicated when there is an acute event or insult that is anticipated to influence test performance, such as major surgery, acute illness, or intervention. The cohort examined in this observational study provides a particularly instructive example of this challenge. It concerns older adults undergoing major surgery with cognitive test performance data assessed immediately prior to and for some time after surgery. Patients’ observed cognitive performance over time is expected to be influenced by retest effects, and also the combined effects of their baseline cognitive abilities, individual variations over time, older age, major surgery (including hospitalization, anesthesia, psychoactive medications, postoperative complications), and in some cases, delirium, an acute confusional state that is common after surgery in older adults [7]. Both surgery [8–10] and specifically delirium [11–14] have been shown to be associated with acute and long-term cognitive decline.

Our goal in this study was to evaluate the use of different methods of correcting for the effects of practice or retest on repeat test administration in the context of an observational study of older adults undergoing elective surgery. It is important to point out that our objective is related to but different from methods of characterizing change using reliable change indices. We are specifically interested in assessing change when there are more than two repeated observations and with investigating the impact of an acute insult or exposure on short- and long-term cognitive change. We first examined the raw data without retest correction, then applied three methods of retest correction that have been utilized in previous studies. Two retest correction approaches, mean difference correction [15] and predicted difference correction [16–18], rely on retest-adjusted cognitive scores based on performance in a non-surgical comparison group. A model-based correction [19, 20] in which the data from the non-surgical comparison group was modeled directly as a reference group was also assessed. We contrast results and inferences from the four approaches with regard to overall trends and the differences in short-term (e.g., 1–2 months) and longer-term (e.g., 6–18 months) cognitive change in a group of patients who developed post-operative delirium compared to those who did not. The objective of this study was to compare the different retest correction methods and to provide insights on strengths and weakness of different methods for future longitudinal studies of older adults employing serial cognitive testing in the setting of acute insults, such as surgery, hospitalization, acute illness, or delirium.

## Materials and methods

### Study populations

We examined data from the Successful Aging after Elective Surgery (SAGES) study cohort along with a non-surgical comparison (NSC) sample measured at the same time [21, 22]. Written informed consent was obtained from all participants according to procedures approved by the institutional review boards of Beth Israel Deaconess Medical Center and Brigham and Women’s Hospital, the two study hospitals, and Hebrew SeniorLife, the study coordinating center, all located in Boston, Massachusetts. The institutional review boards approved this study and all mandatory laboratory health and safety procedures were complied with in the course of conducting this research.

#### Surgical sample (SAGES)

SAGES is an ongoing prospective cohort study of older adults without dementia undergoing major elective surgery. The study design and methods have been described in detail previously [21, 22]. In brief, eligible participants were age 70 years and older, English speaking, scheduled to undergo elective surgery at one of two Harvard-affiliated academic medical centers and with an anticipated length of stay of at least 3 days. Eligible surgical procedures included: total hip or knee replacement, lumbar, cervical, or sacral laminectomy, lower extremity arterial bypass surgery, open abdominal aortic aneurysm repair, and open or laparoscopic colectomy. Exclusion criteria were evidence of dementia, delirium (within 12 months), hospitalization within 3 months, terminal condition, legal blindness, severe deafness, history of schizophrenia or psychosis, or history of alcohol abuse or withdrawal. A total of 566 patients met all eligibility criteria and were enrolled between June 18, 2010 and August 8, 2013. Six subjects were excluded after enrollment due to suspected dementia, determined by neuropsychological testing and clinical review by an expert multi-disciplinary panel, leaving a final sample of 560 participants.

#### Non-surgical comparison (NSC) sample

The NSC sample (*N* = 119) was recruited concurrent to the SAGES sample to evaluate the cognitive trajectory over time in the absence of hospitalization, surgery, and delirium and specifically to quantify retest effects. The approximate size of the NSC sample was selected to provide sufficient precision to quantify the magnitude of retest effects, and is comparable to other studies [23]. NSC patients were enrolled from Beth Israel Deaconess Medical Center [13]. Other than surgery, they met the same inclusion and exclusion criteria set for the surgical group. One subject was excluded after enrollment due to suspected dementia, leaving a final sample of 118.

### Data collection

Surgery participants underwent baseline assessment within 30 days before surgery (median, 9 days) and daily delirium assessment during hospitalization (detailed below). After discharge, participants were followed at 1, 2, 6, and 12 months, and every 6 months thereafter. Our analysis is limited to cognitive test performance data observed over 18 months following surgery. NSC participants were administered the same neuropsychological battery as the surgical sample at a baseline assessment and 1, 2, 6, and 18 months (no 12-month assessment due to logistical constraints). Limited data was collected from their medical record to assess overall level of comorbidity. Delirium was not assessed in the NSC sample.

### Measurement

The following patient characteristics were assessed at baseline in all study groups: age at study intake, sex, race and ethnicity (proportion non-white/Hispanic vs. white/non-Hispanic), marital status, years of education, Modified Mini-Mental State (3MS) examination score (range 0–100; ≤88 indicates cognitive impairment for those with ≥9 years of education) [24, 25], general cognitive performance (GCP) at baseline (described below), Informant Questionnaire on Cognitive Decline in the Elderly (IQCODE; range 1–5, < 3 indicates cognitive decline) [26], Geriatric Depression Scale (GDS; range 0–15, > 5 indicates mild-severe depression) [27], Activities of Daily Living (ADL) impairment (range 0–14, > 0 indicates impairment), Instrumental ADL (IADL) impairment (range 0–14, > 0 indicates impairment), a physical impairment composite score [28] (mean of 50 and standard deviation, SD, of 10; lower scores on the physical composite indicate worse physical functioning and a score of < 35 has been shown to be associated with adverse clinical outcomes), visual impairment (< 20/70 corrected binocular vision), hearing impairment (< 6/12 on Whisper Test) [29], and body mass index (based on self-reported height and weight).

#### Delirium

Post-operative delirium assessment was measured in the surgical cohort using daily brief cognitive testing [22, 30], Delirium Symptom Interview (DSI) [31], and the Confusion Assessment Method (CAM) [31], a standardized delirium rating approach with high sensitivity (94–100%), specificity (90–95%) [32, 33], and inter-rater reliability (kappa statistic = 0.92 in 71 paired ratings in SAGES). To maximize sensitivity for detection of delirium, the CAM results were augmented with a validated chart review method [34, 35].

#### General cognitive performance

A complete neuropsychological test battery was completed at baseline and each follow-up interview, which included: Trail-Making Tests A and B, Phonemic F-A-S Fluency, Category Fluency, Visual Search and Attention Test, Hopkins Verbal Learning Test-Revised, Digit Span Forward/Backward, Boston Naming Test, and Repeatable Battery for the Assessment of Neuropsychological Status Digit Symbol Substitution. From this battery, we created a composite summary measure, the General Cognitive Performance (GCP), which was used as our primary outcome measure of longitudinal cognitive decline. GCP is a weighted composite measure that is calibrated to a nationally representative sample of adults ≥70 years, in order to yield a mean score of 50 and standard deviation (SD) of 10 [36, 37] at the U.S. population level. The GCP is sensitive to change, has minimal floor and ceiling effects, and has been utilized in many prior studies to date [13, 38–40].

### Data analysis

Linear mixed effects (LME) models were used to model baseline levels of and longitudinal changes in cognitive test scores, and to assess differences between those who developed delirium and those who did not in terms of both short- and longer-term cognitive performance. We selected an LME model to measure longitudinal change because we assumed the outcome variable (GCP) to be continuous and for cognitive decline to follow a linear trajectory after the second month. Specifically, we used LME models with maximum likelihood parameter estimation and an unstructured covariance using either the surgical cohort only (*N* = 560) or the surgery + NSC cohorts (*N* = 678). Conditional models included a random intercept, random time-slope from 2 to 18 months, fixed time indicator variable for months 1 and 2, a fixed time-slope from 2 to 18 months, fixed effects for covariates (mean-centered age, female sex, nonwhite race, and years of education), main effect of delirium group, and interactions between time indicator variables and delirium group (1 m × group and 2 m × group) and time-slope and delirium group (2-18 m × group). Delirium group is defined with two levels—surgery delirium-negative (delirium-, reference group) and surgery delirium-positive (delirium+)—for Approaches 1, 2, and 3. For Approach 4, delirium group is defined with three levels—NSC (reference group), surgery delirium-, and surgery delirium+. The four methods for retest correction are described below and summarized in Table 1. The coefficient of determination (*R*^*2*^) was calculated as the squared correlation between the observed and predicted GCP values for each person on each occasion of measurement from each model [41]. *R*^*2*^ is reported for the overall model and within each group. All models assumed that incomplete data were missing at random.

**Table 1:** Description of retest correction approaches

Model	Analysis Sample	Retest Correction Method**	Additional Specifications to Basic Model*	Strengths	Weaknesses
1: No correction	SAGES surgery sample (delirium-positive and delirium-negative) *N* = 560	No correction for retest effect	Main effect: delirium group Interactions: time × delirium group Outcome: raw GCP	-Does not make manipulations to observed data -Does not require a control group	-Difficult to separate retest effects from effects due to delirium/surgery
2: Mean difference correction	SAGES surgery sample (delirium-positive and delirium-negative) *N* = 560	Step 1: Calculate mean retest effect in NSC group: $R{(NSC)}_t=\frac{1}{N}\sum \limits_i GCP{(NSC)}_{it}- GCP{(NSC)}_{i0};$ *t* ∈ (1, 2, 6), *i* ∈ 1, 2, …, *N*) Step 2: Calculate retest-corrected scores in surgical group: *GCPRC*_*it*_ = *GCP*_*it*_ − *R*(*NSC*)_*t*_; where *R*(*NSC*)_6_ = *RSC*(*NSC*)_12_ = *RSC*(*NSC*)_18_	Main effect: delirium group Interactions: time × delirium group Outcome: mean difference corrected GCP	-Straightforward, constant (within occasion) transformation for all people -Correction is uncorrelated with any other variable at each time point	-Potential variability due to the fact that precision of retest correction estimation is not accounted for
3: Predicted difference correction	SAGES surgery sample (delirium-positive and delirium-negative), N = 560	Step 1: Estimate linear regression equations in NSC group expressing the dependence of GCP score at follow-up on baseline GCP, centered at the overall mean of the GCP at baseline in the NSC group; The regression is estimated separately for each observation time point (follow-up month) *t* ∈ (1,2,6): $\kern0.75em GCP{(NSC)}_{it}={b}_{0t}+{b}_{1t}\left( GCP{(NSC)}_{i0}-{\overline{GCP(NSC)}}_0\right)+{e}_t$ Step 2: Compute expected GCP performance given the NSC group regression equations: $E{(GCP)}_{it}={b}_{0t}+{b}_{1t}\left({GCP}_{i0}-{\overline{GCP(NSC)}}_0\right)$ Step 3: Calculate retest effect in surgical (SRG) group: *R*(*SRG*)_*it*_ = *GCP*_*it*_ − *E*(*GCP*)_*it*_; *t* ∈ (1, 2, 6) Step 4: Calculate retest-corrected scores in surgical group: *GCPRC*_*it*_ = *GCP*_*it*_ − *R*(*SRG*)_*t*_; *t* ∈ (1, 2, 6,12,18)	Main effect: delirium group Interactions: time × delirium group Outcome: predicted difference corrected GCP	-Retest effects will be more appropriately modeled if a baseline variable is known to predict retest effects	-The degree of correction could differ by group if predictors of the retest effect differ by group -Inconsistent literature on variables predicting retest effect -Variability due to precision of retest correction estimation is not accounted for in the model
4: Model-based correction	SAGES surgery sample (delirium-positive and delirium-negative), *N* = 560 & non-surgical comparison (NSC) sample, *N* = 118	Raw GCP is utilized for both the NSC and surgical groups, but this method differs from Model 1 (no correction) in that NSC data is modeled as the comparison group for both the delirium+ and delirium- groups. Relative differences between the delirium+ and delirium- groups are then calculated with post hoc tests.	Main effect: NSC, delirium group Interactions: time × group Outcome: raw GCP	-Standard errors are appropriately conservative -Can model differences compared to another surgical group and a NSC sample	-Model estimates are reported in reference to the NSC group -Potential analyses are restricted to variables observable in the NSC group -Maintaining a NSC group is expensive and may introduce additional bias over time due to drop out or latent differences between the NSC and surgical groups.

SAGES=Successful AGing after Elective Surgery; NSC = non-surgical controls; GCP = general cognitive performance

*Basic model: Linear mixed effects model with random intercept and time piece from 2 to 18 months; fixed time indicator variables for months 1 and 2 and time piece from 2 to 18 months; fixed covariates: baseline age, sex, non-white race

**The Approach 2 Step 1 equation defines the normative retest or practice effect [R(NSC)] as the mean difference in general cognitive performance (GCP) score from baseline among the non-surgical comparison (NSC) group. This effect is computed assuming no true change occurs within a six month time frame, or is vanishingly small relative to the practice or retest effect. The retest effect is simply the mean within-person difference between the time *t* follow-up and baseline observed cognitive test score (GCP). These are computed for months 1, 2 and 6 (per-protocol observation time points). Step 2 defines the retest corrected cognitive performance score (GCPRC) for a person (*i*) at time *t* as their observed score at time *t* minus the mean retest effect in the NSC group at time *t*. We set the 12 and 18 month follow-up equal to the six-month retest effect to reflect our assumption that practice or retest effects are constant following the six month follow-up

Previous studies have shown the greatest retest gains between the first and second test administrations [42, 43], with diminishing practice effects leveling off after about 3–4 assessments [3, 44]. While retest effects may last for up to 7 years [1], at least one study has shown that with frequent serial testing, retest effects tend to diminish after about three months across multiple cognitive domains [43]. Moreover, the assumption that changes observed over time reflect primarily the effect of repeated test exposure and minimally reflect maturational changes is justified for only relatively short periods. Therefore, the level of retest correction applied in this study varies for follow-up assessments at months 1, 2, and 6. The same level of correction at month 6 (the fourth assessment) is applied to all subsequent assessments, when retest effects are expected to level off.

Group differences between the surgical and NSC groups were assessed with *t*-tests for continuous variables and chi-square test for dichotomous variables.

#### Approach 1: No retest correction

Approach 1 does not apply any retest correction. Raw GCP is the independent variable and analysis was limited to the surgical sample (*N* = 560).

#### Approach 2: Mean difference correction

This approach was used in the International Study on Postoperative Cognitive Dysfunction (ISPOCD) [15]. The approach first subtracts the observed baseline GCP score from the GCP score at each time point in both the NSC and surgical samples. Then, the mean difference in the NSC sample is subtracted from the observed difference scores seen in the surgical sample at matching time points.

This correction shifts the distribution of the repeatedly observed cognitive performance scores across the entire cohort, but does not impact the rank order of persons or inter-individual variation at any visit. Within occasion, the correction is a constant and therefore uncorrelated with any variable under study. The ISPOCD method also allows for division of the corrected score by the SD of the NSC group mean to create a unitless score, but to preserve comparability of methods we have not done so here. Additionally, to readily compare approaches and maximize interpretability, we returned the corrected value to the GCP scale by adding the individual’s baseline GCP score to the difference score. The mean difference retest-corrected GCP score was then used as the dependent variable in the LME model to test differences between the delirium+ and delirium- groups in the surgical cohort (*N* = 560).

The 6-month assessment in the comparison sample is used as the centering point for the 6 month and all subsequent observations in the surgical sample. This approach relies upon the assumption that differences in the mean across the repeat performances in the comparison sample represent the mean practice or retest effect free of normative cognitive change. We considered this assumption reasonable given evidence from the literature (described in section “Data analysis”) and the relatively short time interval between assessments in this stable outpatient comparison group.

#### Approach 3: Predicted difference correction

The predicted difference method is a regression-based approach that uses a patient’s baseline performance to predict what his/or her retest score is expected to be at retest, using a regression equation derived from a reference sample [16–18]. First, a series of simple linear regressions were performed in the NSC sample to derive regression equations for prediction of GCP performance at months 1, 2, and 6 (dependent variable) based on baseline GCP performance (independent variable). No other variables were included in these initial models. Next, the estimated regression coefficients were used to generate predicted GCP performance at months 1, 2, 6, 12, and 18 for each individual in the surgical sample (Additional file 1: Table S1). The model estimated for month 6 in the NSC group was used to generate expected scores for month 6 onward in the surgical sample. The predicted GCP score was then subtracted from the observed GCP score to derive the retest effect. Finally, an individual’s retest effect was added to his or her observed baseline GCP score to calculate the predicted difference retest-corrected GCP at each visit. These retest-corrected GCP scores were then used as the dependent variable in the LME model to test differences between delirium+ and delirium- groups in the surgical cohort (*N* = 560).

#### Approach 4: Model-based correction

Rather than using the NSC group to derive a retest-corrected GCP score, the model-based approach uses the NSC group as the reference group in the LME model [19, 20]. Raw GCP scores were the independent variable and both surgical and NSC cohorts were combined before analysis (*N* = 678). This approach also uses an interaction between group and time, but in this analysis there are three groups: NSC, surgery delirium-, and surgery delirium+. The coefficients of the surgery delirium- and surgery delirium+ groups were compared with those in the NSC group. Post hoc tests were performed to test if the delirium+ group differed from the delirium- group at months 1 and 2, and whether their slopes differed from months 2–18.

## Results

### Sample characteristics

Baseline sample characteristics are summarized in Table 2. The surgical and NSC groups had nearly identical mean age and baseline GCP scores. Compared to the surgical group, the NSC group had a smaller proportion of females (*χ*^*2*^ = 7.9, *p* = .01), a greater proportion of individuals of non-white race or Hispanic ethnicity (*χ*^*2*^ = 4.6, *p* = .03), more years of education (*t* = 3.8, *p* < .001), lower scores on the Geriatric Depression Scale (*t* = − 5.0), *p* < .001), fewer ADL difficulties or dependency (*t* = − 5.2, *p* < .001), fewer total IADL impairments (*t* = − 3.8, *p* < .001), better physical function (*t* = 11.9, *p* < .001), and lower body mass index (*t* = − 2.8, *p* = .01).

**Table 2:** Sample characteristics at baseline by group

Baseline sample characteristic	Non-surgical comparison (*N* = 118)	Successful Aging after Elective Surgery		
		Total (*N* = 560)	Delirium- (*n* = 426)	Delirium+ (*n* = 134)
Age – years, mean (SD)	77 (5.2)	77 (5.2)	76 (5.2)	77 (5.0)
Sex – n (%) female	52 (44%)	326 (58%)	245 (58%)	81 (60%)
Race/ethnicity – n (%) non-white or Hispanic	16 (14%)	37 (7%)	28 (7%)	9 (7%)
Marital status – n (%) married or living with partner	73 (62%)	332 (59%)	253 (59%)	79 (59%)
Education – years, mean (SD)	16 (3.2)	15 (2.9)	15 (2.9)	15 (3.0)
3MS Score (0–100) – mean (SD)	93.4 (5.6)	93.5 (5.4)	94.1 (5.1)	91.6 (5.8)
Baseline GCP – mean (SD)	58.1 (9.7)	57.6 (7.2)	57.5 (7.3)	54.7 (6.5)
IQCODE (0–5) – mean (SD)*	3.1 (0.19)	3.1 (0.19)	3.1 (0.16)	3.1 (0.25)
Geriatric Depression Scale (0–15) – mean (SD)*	1.3 (1.76)	2.5 (2.5)	2.3 (2.4)	3.0 (0.3)
Total ADL impairment (0–14) – mean (SD)	0.02 (0.16)	0.81 (1.6)	0.75 (1.5)	1.0 (1.9)
Total IADL impairment (0–14) – mean (SD)	0.12 (0.53)	0.54 (1.18)	0.45 (1.1)	0.78 (1.5)
Physical impairment composite – mean (SD)	50.6 (8.0)	38.7 (10.3)	39.3 (10.3)	36.8 (10.0)
Visual impairment – n (%)*	1 (< 1%)	3 (< 1%)	1 (< 1%)	2 (< 2%)
Hearing impairment – n (%)*	39 (33%)	182 (33%)	133 (31%)	49 (37%)
Body mass index – mean (SD)*	27 (4.6)	29 (5.5)	28.3 (5.6)	29.3 (5.2)

Continuous variables are presented as mean (SD) and dichotomous variables are presented as n (%). Successful Aging after Elective Surgery (SAGES) is grouped as the total cohort sample, the group who did not have delirium (Delirium-) and the group who developed delirium (Delirium+). Body mass index is calculated from self-reported height and weight. Visual impairment = < 20/70 corrected binocular vision. GCP = general cognitive performance; 3MS = Modified Mini Mental State Examination; IQCODE = Informant Questionnaire on Cognitive Decline in the Elderly; ADL = Activities of Daily Living; IADL = Instrumental Activities of Daily Living

*Indicates some missing data for the SAGES surgical and/or NSC samples: 2 missing Geriatric Depression Scale, 1 missing IQCODE, 8 missing body mass index (2 from NSC), 1 missing hearing impairment, 3 missing visual impairment

### Overall cognitive trends

Visual examination of our raw data (Fig. 1 displays raw time-series data for a random sample of individuals) showed a consistent pattern: the greatest gains in the NSC group were observed between baseline and the second assessment at month 1, and smaller gains were observed between the second and third assessment, leveling off after month 2. This is consistent with previous studies showing little to no retest effect gains after the 3rd assessment and further justifies our decision to apply a variable level of retest correction only up to month 6 and the same level of correction to all subsequent assessments.

**Fig. 1:**
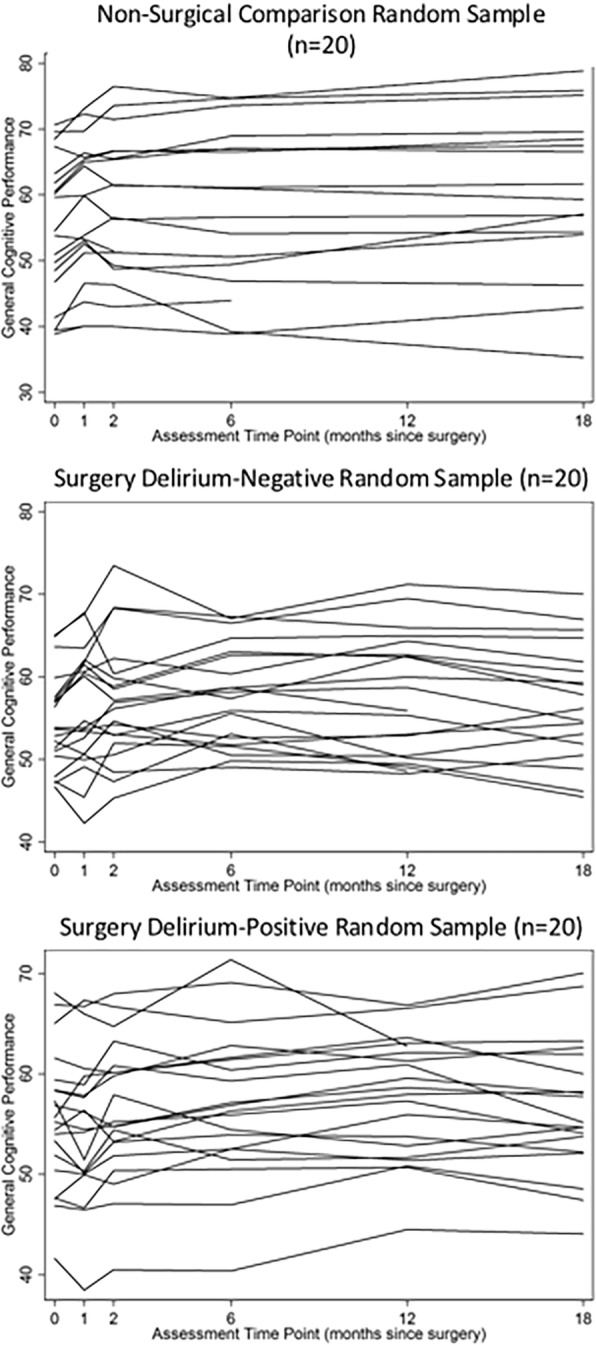
Spaghetti plots of raw general cognitive performance over time. Spaghetti plots of random samples (*n* = 20) per group of raw general cognitive performance (GCP) over time in the non-surgical comparison group (top panel), surgery delirium-negative group (middle panel), and the surgery delirium-positive group (bottom panel). Raw time-series data for all three groups generally show a plateauing of cognitive performance after month 6, with the most variability in the delirium group

GCP performance for both groups started at about the same level, on average, and the mean GCP scores increased at the 1 and 2 month observations and remained stable thereafter (modeled data from uncorrected GCP scores is displayed in Fig. 2). The gain from baseline to month 6 was, on average, 2.6 GCP points in the surgical group and 2.3 GCP points in the NSC group. The GCP is calibrated to have a SD of 10 in a representative sample of U.S. community dwelling elders aged 70 and older, and therefore this performance gain from baseline to 6 months describes a small effect size. The mean score was different between the surgical and NSC groups only at the 1-month assessment (1.04 GCP points lower in the surgical group, *p* = .002).

**Fig. 2:**
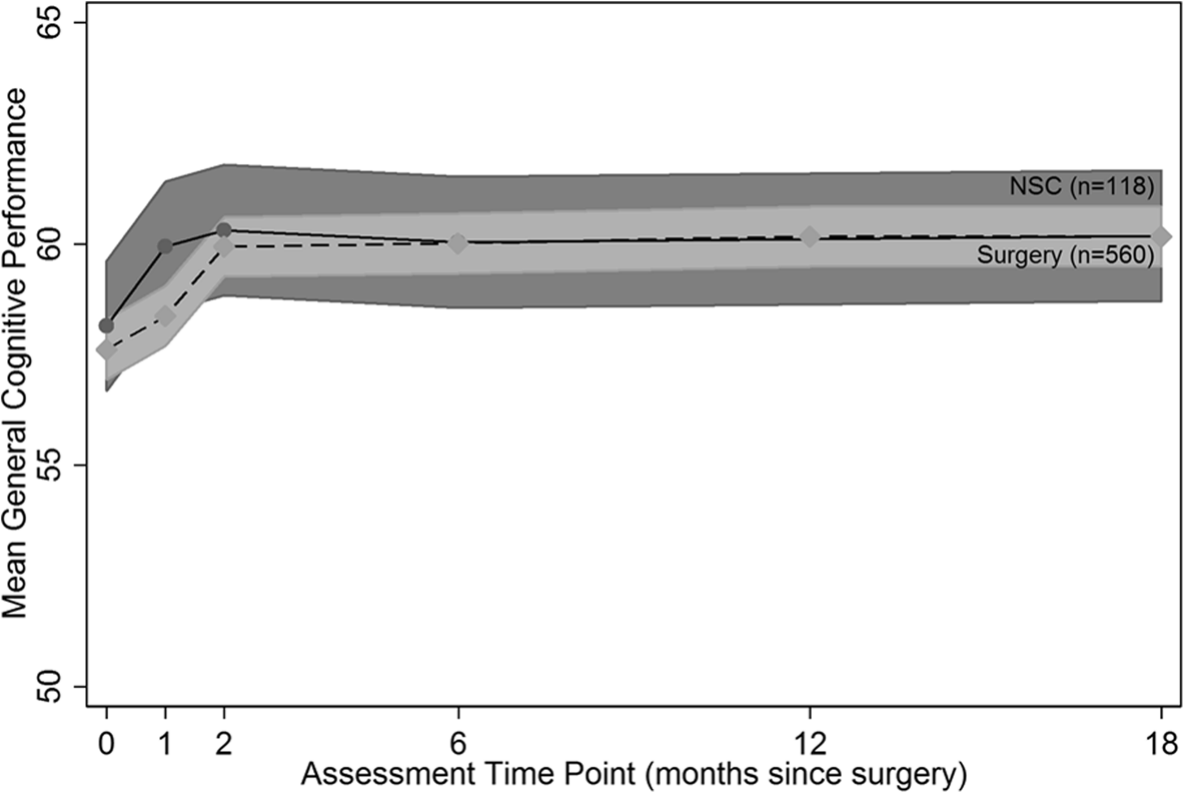
Mean general cognitive performance (GCP) in the surgical vs. non-surgical comparison (NSC) groups. Raw GCP performance in the surgery group (*n* = 560, dashed line, 95% C.I.s in light gray) and NSC group (*n* = 118, solid line, 95% C.I.s in dark gray) estimated using marginal means derived from a linear mixed effects model with random intercept and fixed effects for assessment time points coded as dichotomous “dummy” variables, surgery vs. NSC dichotomous grouping variable, and no additional covariables. X-axis: assessment time point (months since surgery); Y-axis: model-estimated GCP performance

### Comparison of approaches

The parameter estimates and model fit for each retest correction method are summarized in Table 3 and Fig. 3. Overall, we found remarkable consistency in estimates of mean fit and group differences in means; however some differences – particularly in Approach 3 – were also apparent. All approaches indicated that the delirium+ group exhibited greater mean decline than the delirium- group in GCP at month 1, but that this effect was somewhat attenuated at month 2: net differences in GCP scores between the delirium+ and delirium- groups measured by the four approaches differed at baseline from − 2.9 to − 3.0 GCP points, change from baseline to month 1 from − 1.3 to − 1.6 GCP points, and change from month 1 to month 2 from 0.94 to 1.14 GCP points. Estimated time-slopes from 2 to 18 months did not differ substantially between the delirium- and delirium+ groups (difference in slopes ranged from − 0.32 to − 0.34 GCP points per year). Standard errors, model *R*^*2*^ (ranging from 0.33 to 0.37), and *R*^*2*^ by group (ranging from 0.31–0.32 in the delirium- group and 0.25–0.27 in the delirium+ group) were also highly consistent across approaches.

**Table 3:** Comparison of model outputs by retest correction method

	Model 1: No Correction			Model 2: Mean Difference Correction			Model 3: Predicted Difference Correction			Model 4: Model-based Correction			
Model Parameter	Del-	Del+	Net Diff.	Del-	Del+	Net Diff.	Del-	Del+	Net Diff.	NSC	Del-	Del+	Net Diff.
Intercept	58.32 (0.31)	55.33 (0.55)	−3.00* (0.63)	58.32 (0.31)	55.33 (0.55)	− 3.00* (0.63)	58.31 (0.32)	55.35 (0.57)	−2.97* (0.65)	57.86 (0.61)	58.32 (0.31)	55.41 (0.56)	−2.91* (0.64)
Change at month 1	1.08 (0.16)	−0.20 (0.28)	−1.28* (0.32)	−0.73 (0.16)	−2.01 (0.28)	−1.28* (0.32)	−0.67 (0.16)	−2.27 (0.28)	−1.60* (0.32)	1.80 (0.29)	1.08 (0.15)	−0.20 (0.28)	−1.28* (0.31)
Change at month 2	1.39 (0.14)	2.33 (0.26)	0.94* (0.29)	1.08 (0.14)	2.02 (0.26)	0.94* (0.29)	1.04 (0.14)	2.17 (0.26)	1.14* (0.29)	0.32 (0.27)	1.39 (0.14)	2.33 (0.25)	0.94* (0.29)
Change from months 2–18	0.23 (0.12)	−0.11 (0.22)	− 0.34 (0.25)	0.40 (0.12)	0.06 (0.22)	−0.34 (0.25)	0.39 (0.12)	0.07 (0.22)	−0.32 (0.25)	−0.10 (0.27)	0.23 (0.12)	−0.11 (0.22)	− 0.34 (0.25)
Model *R*^*2*^ by group	0.32	0.27		0.31	0.25		0.31	0.25		0.47	0.32	0.27	
Model *R*^*2*^	0.35			0.33			0.33			0.37			

Comparison of model outputs for baseline GCP (intercept), change in GCP from baseline to 1 month, change in GCP from month 1 to 2, and estimated GCP slope from 2 to 18 months by retest correction method. Model outputs are presented as parameter estimates (and standard errors) for intercept, time indicator variables at months 1 and 2, and time-slope from months 2–18 by group for each retest correction method

Del- = surgery Delirium-negative group (*n* = 426), Del + = surgery delirium-positive group (*n* = 134), NSC = non-surgical comparison group (*n* = 118). GCP = General Cognitive Performance. For models 1–3, Del- is the reference group; for model 4, NSC is the reference group. Net diff = net difference calculated as the β-coefficient in the surgery delirium- group subtracted from the β-coefficient in the surgery delirium+ group. *R*^*2*^ is the squared correlations of observed and model-implied outcome values

*Indicates a significant difference between delirium groups at α = 0.05

**Fig. 3:**
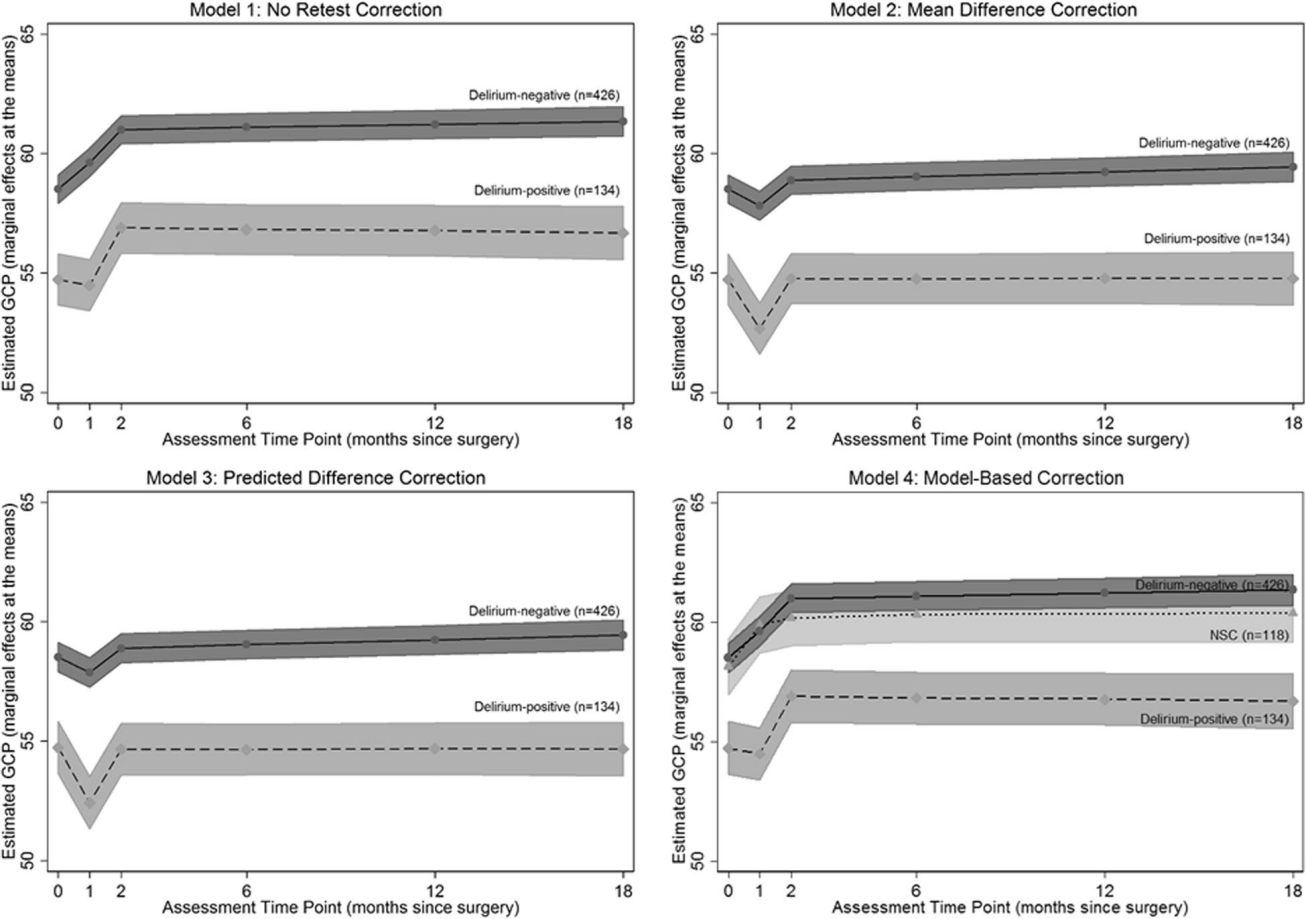
General cognitive performance (GCP) over time using the four retest correction methods. Models 1-3) SAGES surgical sample by delirium group: Delirium-negative (*n* = 426, solid line, 95% C.I.s in dark gray) and Delirium-positive (*n* = 134, dashed line, 95% C.I.s in medium gray); Model 4) SAGES surgical sample by delirium group and non-surgical comparison (NSC) sample (*n* = 118, dotted line, 95% C.I.s in light gray). X-axis: assessment time point (months since surgery); Y-axis: model-based estimated GCP at the means of covariates (age = 77 years, 56% female, 1% nonwhite race/ethnicity, education = 15 years)

Figure 4 and Table 4 display the average GCP performance at each time point in the delirium+ and delirium- groups using raw (uncorrected) GCP (used in Approaches 1 and 4), mean difference-corrected GCP (used in Approach 2), and predicted difference-corrected GCP (used in Approach 3). While relative differences between the delirium- and delirium+ groups were mostly unaffected by retest correction, the model-implied GCP scores and corresponding evidence of cognitive change varied by method. For instance, in Approach 1 (no correction) and Approach 4 (model-based correction), which used raw GCP data, it appears that the delirium- and delirium+ groups improved or remained stable one month after surgery and continued to improve at month 2. Although the standard errors (SE) and confidence intervals (CI) differ slightly, the estimates are identical: raw scores for both Approach 1 and Approach 4 indicate that the delirium- group improved by 1.08 GCP points, while the delirium+ group declined slightly by − 0.20 GCP points. The delirium- group improved from month 1 to 2 by an additional 1.39 GCP points and the delirium+ group improved by 2.33 GCP points. In other words, raw GCP scores indicate that surgical patients will improve by 2 or more GCP points, equivalent to more than a fifth of a population SD, two months after surgery. Comparable increases in the NSC group (increases of 1.80 GCP points at month 1 and an additional 0.32 at month 2) strongly indicate that these perceived improvements are due to factors unrelated to surgery, and demonstrate the importance of correcting for retest effects if model-implied means will be evaluated in addition to relative differences.

**Fig. 4:**
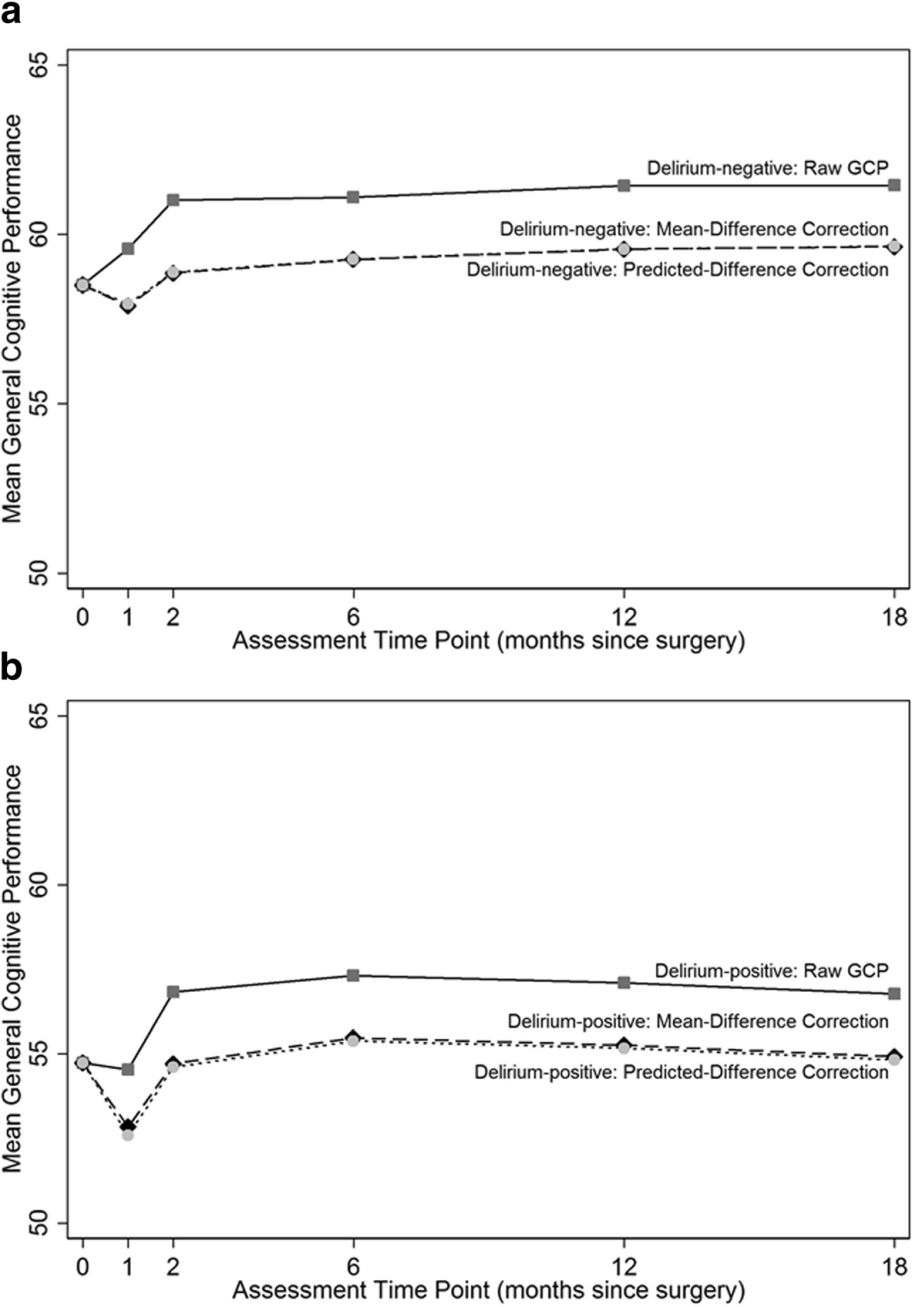
Absolute mean general cognitive performance by delirium status. Absolute mean general cognitive performance (GCP) in the (**a**) Delirium-negative and (**b**) Delirium-positive groups using raw/uncorrected GCP (dark gray squares, used in Models 1 and 4), mean difference-corrected GCP (black diamonds, used in Model 2), and predicted difference correction (light gray circles, used in Model 3). X-axis: assessment time point (months since surgery); Y-axis: mean GCP performance. Means and standard deviations for each time point by group are provided in Table 4

**Table 4:** Raw and corrected mean (standard deviation) GCP at each visit by group

	NSC			Delirium-Negative			Delirium-Positive		
Visit	Raw GCP	Mean Difference-Corrected GCP	Predicted Difference-Corrected GCP	Raw GCP	Mean Difference-Corrected GCP	Predicted Difference-Corrected GCP	Raw GCP	Mean Difference-Corrected GCP	Predicted Difference-Corrected GCP
Month 0	58.14 (9.74)	N/A	N/A	58.51 (7.29)	58.51 (7.29)	58.51 (7.29)	54.73 (6.49)	54.73 (6.49)	54.73 (6.49)
Month 1	59.60 (9.30)	N/A	N/A	59.58 (7.82)	57.90 (7.75)	57.96 (8.3)	54.54 (7.07)	52.84 (6.97)	52.58 (7.43)
Month 2	60.20 (9.64)	N/A	N/A	61.01 (7.8)	58.86 (7.77)	58.88 (7.98)	56.82 (7.44)	54.72 (7.36)	54.61 (7.53)
Month 6	60.49 (9.68)	N/A	N/A	61.10 (7.51)	59.26 (7.42)	59.26 (7.59)	57.31 (7.29)	55.48 (7.18)	55.38 (7.32)
Month 12	N/A	N/A	N/A	61.44 (7.68)	59.56 (7.64)	59.56 (7.81)	57.10 (6.55)	55.26 (6.43)	55.17 (6.56)
Month 18	60.59 (9.71)	N/A	N/A	61.46 (7.85)	59.64 (7.77)	59.64 (7.93)	56.77 (7.7)	54.92 (7.64)	54.82 (7.79)

This table contrasts the mean absolute scores of the raw and retest-corrected General Cognitive Performance (GCP) across the three clinical groups of interest: non-surgical comparison group (NSC; *n* = 118), surgical patients who did not develop delirium (Delirium-negative; *n* = 426), and surgical patients who developed post-operative delirium (Delirium-positive, *n* = 134). Approaches 1 and 4 both utilized raw GCP data; however, Approach 1 only compared the delirium-positive and delirium-negative groups while Approach 4 first compared both surgical groups to the NSC group and subsequently performed post hoc tests to compare the delirium-positive and delirium-negative groups. Approach 2 utilized mean difference-corrected GCP and Approach 3 utilized predicted difference-corrected GCP

In contrast, Approach 2 (mean-difference) and Approach 3 (predicted-difference correction), which dissociate retest and surgery effects by using retest-adjusted GCP scores, revealed a decline in performance in both surgical groups at month 1 and a recovery to baseline at month 2. Specifically, in Approach 2, at month 1 the delirium- group declined by − 0.73 GCP points then improved from month 1 to month 2 by 1.08 GCP points; the delirium+ group declined even more markedly at month 1 by − 2.01 GCP points, but recovered at month 2, improving by 2.02 GCP points. Similarly, in Approach 3, the delirium- group declined by − 0.67 GCP points at month 1 but improved from month 1 to month 2 by 1.04 GCP points; the delirium+ group declined at month 1 by − 2.27 GCP points then recovered at month 2, improving by 2.17 GCP points. Slopes from months 2–18 did not differ by approach; this was expected since, similar to previous studies, the largest retest gains are observed after the first two repeated administrations (at months 1 and 2).

## Discussion

Retest effects are a known source of bias in longitudinal studies. Our goal was to contrast results and inferences derived from four existing methods in the literature for addressing practice or retest effects in an observational study of cognitive performance following elective surgery. Overall, we found that all four approaches provided nearly equivalent information and would lead to identical inferences regarding *relative* mean differences between groups experiencing a key post-operative outcome (delirium). Conversely, the three methods produced qualitatively different impressions of *absolute* performance differences over 18 months following surgery: uncorrected GCP scores increased by more than 1/5 of a population SD two months after surgery in both the surgical and NSC groups, suggesting that this increase is primarily due to retest rather than surgery; in contrast, retest-corrected GCP scores revealed a decline in performance in both surgical groups at month 1 and a recovery to baseline by month 2.

Our main finding, that substantive inferences and effect size estimates relating to the relative impact of exposure variables on cognitive changes are robust to different approaches to retest effects, echoes similar findings reported by Vivot et al. (2016) despite those authors considering a different context of study (long-running observational cohort studies of cognitive aging vs. follow-up studies of a clinical cohort), types of exposures (diabetes and depression vs. delirium) and studied approaches to handling practice and retest effects (model-based approaches with no control group vs. data manipulation and model-based approaches with a control group) [20]. Our findings are also congruent with those of Salthouse (2016) [45] who concluded that the primary impact of practice and retest effects was to disrupt mean age trend trajectories in the context of retest effects, whereas slopes over time are relatively unconfounded by retest effects. It is also notable that despite similar conclusions to our study, Vivot et al. and Salthouse used different approaches to quantify practice and retest effects (quasi-longitudinal or sequential cohort design).

Our study combined with those of Vivot et al. and Salthouse, all of which used different practice/retest effect adjustment methods suited to different study designs and research questions, demonstrates that retest effects influence age-related trends but are less important for understanding the relative impact of risk factors. Therefore, correction for retest effects is especially important for descriptive and natural history studies, but for analytical epidemiology studies – for example, characterizing the impact of discrete risk factors (such as delirium, depression, diabetes) – the impact of addressing retest effects is in interpretation and building the narrative to set the context of observed differences and effects of exposures. Of the four approaches examined, only Approach 1 (no correction) contradicts our *a priori* assumption based on clinical observations that surgery causes an acute decline in cognition. Furthermore, Approach 2 (mean difference correction) is the most straightforward method for interpreting cognitive trends since Approach 3 (predicted difference correction) changes the rank order and Approach 4 (model-based correction) requires extra post hoc transformations to generate interpretable results.

There are other important strengths and weakness of each retest correction method that should be considered before choosing the best approach for a particular study (Table 1). The primary strengths of Approach 1 are that it does not make manipulations to observed data and does not require a control group. However, because it is difficult (or impossible) to separate the effects of retest and exposure, it is only appropriate for studies examining relative differences between groups or only in long-term cognitive change, provided that a slope is only fit to data after the first 2–3 administrations, after the most significant retest gains have already occurred. Approach 4 utilizes raw GCP data from the surgical group as well as from the NSC group to model retest effects and variation in retest effects across a population. Although the latter is a strength of this approach, its importance is attenuated by our finding that model fit indices and standard errors were nearly identical across approaches, suggesting that variance in retest effects does not substantially impact inferences. A primary limitation of Approach 4 is that using a NSC group as the reference group in a statistical model restricts the types of hypotheses that can be tested. For instance, surgery type, anesthesia type, or other exposure-related factors cannot be modeled as covariates or predictors of cognitive decline since these variables cannot be collected in patients who are not undergoing surgery. This significantly limits the applicability of this method for many studies.

Rather than modeling the NSC data directly, Approaches 2 and 3 use the NSC data to generate retest-corrected GCP scores. The key difference between Approach 2 and Approach 3 is that Approach 2 uses a constant retest effect correction for every participant while Approach 3 allows the magnitude of the retest effect correction to vary by the participant’s baseline GCP. A primary strength of Approach 3 is that variables that could influence retest effects can be used to predict retest effects on an individual basis. Indeed, various characteristics have been suggested to influence retest effects [46, 47], including baseline cognitive ability [5, 44]. However, this literature is inconsistent, making it difficult to reliably select appropriate prediction variables [46, 48]. Because we did not expect to draw more definitive conclusions about predictors of retest in our sample than prior work, and to keep the four Approaches as consistent as possible for comparison purposes, we chose not to include other potential predictors of retest in Approach 3. However, should consistent drivers of retest emerge, this would be a considerable advantage for using Approach 3 since those variables could be included in the retest prediction model. In building the predictive model for Approach 3, we observed that baseline GCP scores were negatively correlated with change in GCP scores in the NSC sample. The phenomenon of regression to the mean [49, 50] would lead to the expectation that change scores on two variables that are positively correlated and have similar variance (e.g. baseline and follow-up GCP) would be negatively correlated with baseline performance. So, while this observation is expected given the phenomenon regression to the mean, it may also signal limitations of using a linear model to describe the dependence of follow-up scores on baseline scores.

Approach 2 is a relatively straightforward method which uses a constant transformation derived from the NSC group. This transformation is applied uniformly across all participants and is uncorrelated with any other variable. This strength may illustrate the primary reason for differences in the model outputs between Approaches 2 and 3. Approach 3 allows the mean difference correction to vary as a function of a participant’s baseline GCP, but it is known that cognitive performance is correlated with delirium, our independent variable of interest in the present analyses. Approach 2, therefore, is preferred over Approach 3 when the variable of interest is correlated with cognitive performance, as this may result in biased estimates of difference. The primary advantages of Approach 2, mean-difference correction, are its relatively straightforward application, that it enables interpretation of both relative differences and absolute performance, and that the hypothesized limitation that it does not account for variability due to precision of retest correction estimation has a negligible impact on inferences and model fit.

Our study also compared shorter and longer-term cognitive performance between the surgical and NSC groups. The mean score is different across groups only at the 1-month assessment, where the lingering effects of surgery, including postoperative delirium, are presumed to depress scores for some people. It is also worth noting the slightly lower score at baseline, but equivalent score at month 6 and beyond, suggesting the possibility that the performance of the surgical group at baseline might be depressed by factors related to the impending surgery (e.g., stress, pain, use of pain medications), rather than true differences in cognitive abilities. If it is true that surgical cohorts systematically have lower baseline cognitive scores compared to a NSC group, then a retest prediction model derived in the NSC sample based on baseline cognitive scores (as in Approach 3) may create biased predictions when applied to a surgical sample. This potential source of bias should be considered in future studies that plan to use Approach 3 to correct for retest effects.

This study offers an innovative contribution to the study of retest effects because it specifically assesses approaches that are applicable to observational studies with longitudinal cognitive assessment with two or more time-points aimed at investigating the impact of an acute insult or exposure on short- or long-term cognitive change. Indeed, some of the most common methodologies for controlling for retest effects cannot be evaluated using this type of study design. For instance, although many studies have evaluated the “reliable change index” [51–56], these methods are less applicable to studies with more than two assessments. Additionally, “boost” correction [4, 57], which typically uses a step function to model improvement after the first assessment (i.e., using a function of 0–1–1-1…1 across assessment time points), will not work as designed if there are other factors affecting performance at the second assessment besides retest effects. In the present study, surgery occurred in the intervening period between the first and second assessment; thus the retest “boost” would be biased (likely attenuated) by surgery effects. In contrast, the four approaches evaluated herein are appropriate for study populations where the second or third test administration co-occurs with the acute exposure under study.

This study also has several limitations. First, our study was based on an observational cohort study, and does not provide a “gold standard” by which to measure retest effect. Fortunately, all four approaches provided similar estimates of effect and inferences were qualitatively indistinct for our primary point of inference – delirium+ and delirium- group differences in longitudinal trends of cognitive performance. Second, all models examined here assumed that incomplete data were missing at random. It is possible that bias due to non-random drop out affected both our retest effect estimates in the NSC group and modeling of the effect of delirium in the surgical sample. The latter has been investigated in prior work, which found that estimations of long-term decline in SAGES were robust to multiple different assumptions about missing data (see the Supplementary Appendix of reference [13]). In the NSC sample, all participants returned for the second assessment at month 1, five participants (4%) did not return for their third assessment at month 2, and an additional eight participants (11%) did not return for their fourth assessment at month 6. Although all participants returned for the second assessment, when the greatest retest gains are usually observed, it remains possible that drop-outs may have influenced our retest effect estimations. Third, because it remains unclear which variables consistently influence retest effects, it is possible that our findings may not be generalizable to cohorts that are younger, more racially and ethnically diverse, or less educated. Moreover, it is possible that important differences in our NSC group (e.g. greater baseline cognitive performance, more years of education, fewer physical and functional impairments) compared to the surgical cohort impacted our results. However, a recent study of a community-based cohort of older adults (mean age 77, *N* = 4073) found that, similar to other studies [48], retest effects did not differ as a function of individual differences in race/ethnicity, sex, language, years of education, literacy, apolipoprotein E ε4 status, or cardiovascular risk [46]. Fourth, because retest effect may vary by cognitive domain [58], it is possible the optimal retest correction would also vary by cognitive domain. This is an important area for future study. Finally, the retest correction approaches analyzed have been previously studied in various fields and were selected specifically for this study design, but the chosen methods are not an exhaustive list, and it is possible that alternative approaches exist. In fact, a gold standard approach for this type of study might be repeated observations *prior* to the acute event, such that retest effect has been exhausted before the event or insult of interest [44, 59]. However, given that SAGES participants were enrolled in anticipation of an impending surgical procedure, such an approach might not be feasible for other studies as well.

## Conclusion

In conclusion, this study addressed an important question about potential bias due to uncorrected retest effects in observational, longitudinal studies of surgical populations. Our results show that retest correction is critical for interpretation of absolute cognitive performance measured over time and, consequently, for advancing our understanding of the effects of exposures such as surgery, hospitalization, acute illness, and delirium. Each of the four retest correction approaches analyzed in this study has strengths and weakness that should be evaluated in the context of future studies. Performing simulation studies, rather than analyzing observational data, may shed further light on this phenomenon and extend the findings of the present study, and thus is an important future direction.

## Additional file

Additional file 1: **Table S1.** Model parameters for predicting GCP in Approach 3. (PDF 131 kb)

## Abbreviations

3MS: Modified Mini-Mental State
ADL: Activities of Daily Living
CAM: Confusion Assessment Method
CI: confidence interval
DSI: Delirium Symptom Interview
GCP: general cognitive performance
GDS: Geriatric Depression Scale
IADL: Instrument Activities of Daily Living
IQCODE: Informant Questionnaire on Cognitive Decline in the Elderly
ISPOCD: International Study on Postoperative Cognitive Dysfunction
LME: linear mixed effects
NSC: non-surgical comparison
SAGES: Successful Aging after Elective Surgery
SD: standard deviation
SE: standard error
