# Supplementary material for: Correction for retest effects across repeated measures of cognitive functioning: a longitudinal cohort study of postoperative delirium
Source: BMC Med Res Methodol. 2018 Jul 3;18:69. doi: 10.1186/s12874-018-0530-x (PMC6029140; doi:10.1186/s12874-018-0530-x)
Supplement: Supplementary file 1 — Table S1. Model parameters for predicting GCP in Approach 3. (PDF 131 kb) [file 12874_2018_530_MOESM1_ESM.pdf]

Racine et al

Correction for Retest Effects across Repeated Measures of Cognitive Functioning: A Longitudinal Cohort Study of Postoperative Delirium

SUPPLEMENTARY MATERIAL

Supplementary Table 1. Model parameters for predicting GCP in Approach 3

Outcome variable	Slope	Intercept	R^2	Equation for calculating predicted scores (Y'_i)**
GCP at month 1	0.92 [0.88, 0.97]	6.25 [3.52, 8.98]	0.93	$(0.92 \times BaselineGCP_i) + 6.25$
GCP at month 2	0.97 [0.91, 1.02]	4.20 [0.94, 7.45]	0.91	$(0.97 \times BaselineGCP_i) + 4.20$
GCP at month 6*	0.97 [0.91, 1.03]	3.52 [0.04, 7.01]	0.91	$(0.97 \times BaselineGCP_i) + 3.52$

Coefficients with 95% confidence intervals are reported for slope and intercept.
*The equation generated from the model using GCP at month 6 in the NSC sample was used to calculate predicted scores for month 6 onward in the surgical sample.
**The retest effect was calculated as Observed (Y_i) – Predicted (Y'_i) scores. Retest corrected scores were calculated as the observed GCP score (Yi) + retest effect.
